# Perspective: THz-driven nuclear dynamics from solids to molecules

**DOI:** 10.1063/1.4992050

**Published:** 2017-12-22

**Authors:** Peter Hamm, Markus Meuwly, Steve L. Johnson, Paul Beaud, Urs Staub

**Affiliations:** 1 Department of Chemistry, University of Zurich, Zurich, Switzerland; 2 Department of Chemistry, University of Basel, Basel, Switzerland; 3 Institute for Quantum Electronics, ETH Zurich, Zurich, Switzerland; 4 Paul Scherrer Institute, Villigen, Switzerland

## Abstract

Recent years have seen dramatic developments in the technology of intense pulsed light sources in the THz frequency range. Since many dipole-active excitations in solids and molecules also lie in this range, there is now a tremendous potential to use these light sources to study linear and nonlinear dynamics in such systems. While several experimental investigations of THz-driven dynamics in solid-state systems have demonstrated a variety of interesting linear and nonlinear phenomena, comparatively few efforts have been made to drive analogous dynamics in molecular systems. In the present Perspective article, we discuss the similarities and differences between THz-driven dynamics in solid-state and molecular systems on both conceptual and practical levels. We also discuss the experimental parameters needed for these types of experiments and thereby provide design criteria for a further development of this new research branch. Finally, we present a few recent examples to illustrate the rich physics that may be learned from nonlinear THz excitations of phonons in solids as well as inter-molecular vibrations in liquid and gas-phase systems.

## I. INTRODUCTION

Ultrafast pump-probe experiments, which were developed in parallel with short pulse lasers (initially Q-switched and later mode-locked) starting from the early 1960s paved the way for the field of femtochemistry, i.e., the study of real-time dynamics of chemical reactions in molecular systems, for which Zewail was awarded with the Nobel prize in 1999.^1^ Ultrafast pump-probe experiments have also made strong contributions to other areas of the physical sciences, in particular, the study of strongly correlated solid state materials. In a pump-probe experiment, an ultrashort pump pulse of laser light triggers a process, and another ultrashort pulse interacts with the system and measures the transient changes in the structural, electronic, or optical properties after a well-defined time delay. In the vast majority of pump-probe experiments that are performed, both pump and probe pulses only indirectly couple to the many degrees of freedom of the investigated sample. That is, the pump as well as the probe-pulses resonantly excite particular quantum-states of the system under study; in most cases, electronic state transitions in the UV/VIS spectral range and the information about the real-space motion of the electrons and/or nuclei have to be deduced indirectly from the spectroscopic response.

Regarding the probe process, recently new methods have become available that measure the transient response of electrons and/or nuclei directly in real space by ultrafast electron microscopy,^2–4^ and almost equally directly via a Fourier-transformation in ultrafast transient X-ray scattering,^5–28^ electron scattering,^29–32^ photoelectron scattering,^33,34^ or EXAFS^35^ experiments. An equally direct and structurally specific interaction for the pump process, however, has lagged behind significantly.

In principle, pump pulses of electromagnetic radiation that have frequency content in the THz range would partially address this problem and would be very much in the spirit of the original idea of femtochemistry.^1^ Whereas pump pulses in the visible and ultraviolet range of the electromagnetic spectrum interact predominantly with electronic states and exert forces on the nuclei only indirectly, pump pulses with significant spectral power in the THz range can drive electric-dipole active vibrational modes by exerting forces directly on the charged nuclei. The THz pump process does not even have to be truly resonant, in particular, given that the most intense THz pulses^36–38^ are often quasi-single-cycle pulses that have no frequency selectivity but rather excite the system in an impulsive manner. Typical THz photon energies are in the order of *k_B_T* (at room temperature) or below, which implies that many of the excitations accessible to THz frequency radiation are thermally excited degrees of freedom, e.g., the hydrogen-bonds in a liquid like water, which often determine the equilibrium physical and chemical properties of various systems. Despite this, the number of works that use THz pulses to trigger nuclear dynamics directly is still rather limited, particularly for disordered molecular systems. In this Perspective, we discuss the technical challenges and the scientific potential of such experiments for solid-state, liquid, and gas-phase systems. We also will make a strong point of drawing conceptual connections between THz-induced phenomena in these different kinds of physical systems, where often the language applied to describe quite similar dynamics is different since distinct scientific communities are involved. Our aim here is to take advantage of the potential for cross-fertilization between these different communities to help foster nonlinear THz experiments in all areas of physical sciences.

For the most part, we will concentrate on THz-pump experiments where an intense THz pulse drives a structural response that is then observed by another probe process. Examples include THz-pump-X-ray-probe^9–13,20^ or THz-pump-photoelectron-scattering-probe^39^ experiments, which probe the structural response most directly, but the optical response of the system under study, such as its reflectivity, is often measured as well.^40–44^ A review on nonlinear THz spectroscopy can be found in Ref. 45. For solids, where the phonons are harmonic to a very good approximation, the system often responds to the THz field in a mostly linear fashion.^10,12,13^ Ultimately, one is also interested in a nonlinear regime, exploring the anharmonicity of the potential energy surfaces, on which the nuclei move.^18,46–49^ Driving a nonlinear excitation to the extreme, one might even be able to induce a phase-transition with the THz excitation.^10,18,20,46,50,51^ Although it has not yet been realized experimentally, THz excitation could in principle induce a phase transition by coherently driving a set of structural coordinates over an energy barrier; the displacive first order structural transition in BaTiO_3_ could be a good candidate for this.^52^ Another possible method is to use strong mid-infrared excitation of high frequency vibrational modes to induce an effective renormalization of low frequency structural coordinates via anharmonic coupling, ultimately leading to a structural symmetry change.^53^

The ultimate nonlinear THz experiment is 2D-THz spectroscopy, which has the potential to observe the nonlinear response function completely, typically to all orders.^54–60^ In such an experiment, both pump and probe processes act in the THz regime, and the experiment measures the nonlinear component of the system response to the applied THz fields. Such an experiment requires phase stability of both the pump and probe to allow a 2D Fourier-transformation, in which both the pump and the probe process are presented in the frequency domain. In contrast to 2D spectroscopy in the mid-IR and UV/Vis regime,^61,62^ however, the step from THz-pump-THz-probe spectroscopy^63^ to 2D-THz spectroscopy comes basically for free, since the common generation processes for THz pulses render them inherently carrier-envelope-phase stable, and since the common techniques to measure THz pulses by free-space electro-optic sampling^64^ measure the THz field directly without the need for optical heterodyning. 2D-THz spectroscopy has been pioneered by Elsaesser and co-workers, investigating low-lying electronic transitions in various solid state materials,^54–58^ while the first 2D-THz experiment on a molecular system has been published only very recently by Nelson and co-workers.^59^ Review articles on 2D THz spectroscopy can be found in Refs. 65 and 66.

## II. LIGHT-MATTER COUPLING WITH THz PULSES

A THz pulse with electric field strength *E* acts directly on the charges of the electrons or the nuclei of the system under study. The interaction energy of the field with the transition dipole *μ* of the corresponding transition is given by *μΕ*, and both quantities determine how strongly a THz pulse can perturb the system.

### A. Transition dipole of THz transitions in solids and molecules

**FIG. 1.**
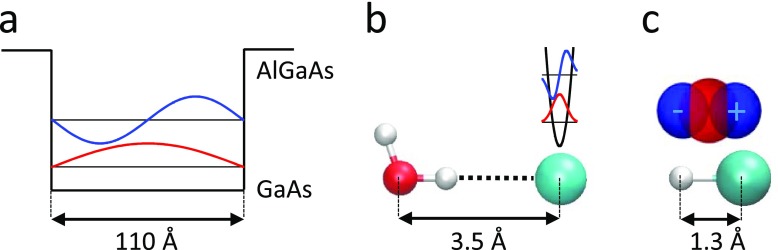
Typical ground- (red) and first excited-state (blue) wavefunctions for (a) a low-lying electronic transition, (b) an inter-molecular vibration, and (c) a rotational transition. In panel (a), this is exemplified for the intersubband transition in a GaAs/AlGaAs quantum well heterostructure with a size of 110 Å,^57^ which can be modelled qualitatively as a particle-in-a-box with the help of *k·p* band structure calculations.^69^ In panel (b), the inter-molecular hydrogen bond vibration between a chloride ion and a water molecule is considered, whose distance and vibrational frequency in liquid water is ∼3.5 Å (Ref. 70) and ∼200 cm^−1^,^71^ respectively. The harmonic wavefunctions have a width of ∼0.12 Å (rmsd.) and are plotted on scale relative to the size of the molecular complex. In panel (c), a HCl molecule (bond length 1.3 Å) is shown together with its rotational eigenfunctions [i.e., the spherical harmonics $Y_{0}^{0}$ and $Y_{1}^{0}$, which are scaled such that the diameter of the ground-state wavefunction (red) matches the HCl bond length]. Panels (b) and (c) are plotted on scale so that the size of the wavefunctions can be directly compared, illustrating the even smaller transition dipole of vibrational transitions.

The transition dipole is defined as $\left\langle i\left|\hat{\mu }\right|j\right\rangle$, where *i* and *j* are the initial and final quantum states, and as such, it is a material or molecular property. The transition dipoles of low-lying, delocalized electronic states in solids are typically huge, which can be understood at least qualitatively from a simple particle-in-a-box picture. That is, a low excitation energy implies a large “box” with the transition frequency scaling as $1/r_{box}^{2}$, which in turn results in a large displacement of charges upon the transition, rendering the transition dipole $\left\langle i\left|\hat{\mu }\right|j\right\rangle$ large. The particle-in-a-box picture is more or less directly transferrable to the problem of intersubband transitions in quantum well heterostructures [see Fig. 1(a)], provided that the width of the well is still well below the wavelength of the THz light.^67^ For example, a transition dipole of 120 D, corresponding to the displacement of a single charge over 25 Å, has been reported for a GaAs/AlGaAs quantum well with a size of 110 Å.^56,57^ The particle-in-a-box argument still holds for other types of electronic transitions in a more qualitative sense, e.g., the transition dipole of the interband transition in graphene is as large as ∼3800 D (*e *×* *800 Å),^54^ that of the ionization of shallow impurities in a doped Ge:Ga semiconductor 320 D (*e *×* *67 Å),^68^ and that of the interband transition in InSb 190 D (*e *×* *40 Å).^55^

When talking about molecules, on the other hand, the degrees of freedom in the THz spectral range are either rotations (only in the gas phase) or vibrations (both liquid and gas phase); in the latter case, these are mostly inter-molecular vibrations between two molecules that are bound by interactions much weaker than typical covalent bonds, e.g., hydrogen bonds. Low-lying electronic transitions would be exceptional for molecular systems, according to the particle-in-a-box argument given above; most molecules are just too small for that to happen. The transition dipole for the excitation of one vibrational quantum is on the order of only ∼0.5 D (*e *×* *0.1 Å) even for a very polar vibrator. This value is estimated from a typical oscillation amplitude of ∼0.1 Å of nuclei that may have partial charges on the order of 1 *e*, a situation which is exemplified in Fig. 1(b) for the inter-molecular vibration of a hydrogen-bonded H_2_O·Cl^−^ complex in liquid water. Rotational transitions are larger by roughly one order of magnitude (∼5 D or *e *×* *1 Å), since it is the size of the molecule as a whole (a few Å), and not the oscillation amplitude, that counts for the transition dipole, see Fig. 1(c) for an HCl molecule.

Optical phonons in solids, which are an important target of nonlinear THz studies as well,^10–13,41,42,46^ bridge the gap between solids and molecules. The molecular-physics counterpart of optical phonons are normal modes in molecules with high symmetry or vibrational excitons in molecular chains such as proteins.^62^ In either case, these vibrations are delocalized to a certain extent, where the degree of delocalization is determined by the symmetry of the system, the domain sizes, the amount of disorder (Anderson localization),^72^ and the anharmonicity. The relative magnitudes of these effects differ strongly in solids vs. molecular systems. Crystalline solids are translationally invariant over very long (macroscopic) length scales and the amount of disorder is typically very small in comparison to molecular systems. According to Bloch's theorem, the harmonic normal modes (phonons) in a perfectly translationally invariant system are infinitely delocalized. In a real crystal, phonons have a finite mean free path due to boundary effects, scattering from impurities, and scattering from other phonons via anharmonic interactions. In the limit of a perfect crystal, the transition dipole of a delocalized vibration scales as $\sqrt{n}\mu_{0}$, where *μ*_0_ is the transition dipole of the corresponding localized vibration (i.e., the unit cell in a solid or a single bond in a molecule) and *n* is the number of contributing vibrations in a delocalized state, i.e., the delocalization volume. This means that the transition dipole for optical phonons in crystals is typically very high in comparison to the corresponding transition dipole for a single unit cell, since typical phonon mean free paths are many times larger than a lattice constant. Delocalization is also responsible for the fact that vibrations become more harmonic in comparison to the corresponding localized vibration. The anharmonicity of a delocalized vibration, defined as the difference of the fundamental frequency between ground and first excited state and that between first and second excited state, scales as 1/*n*.^73^ In simple words, the lowering of the anharmonicity can be understood by the fact that the oscillation amplitude of the atoms decreases by $1/\sqrt{n}$ as the delocalization volume *n* increases, forcing the atoms to explore less of the potential energy surface. Both properties, the essentially infinite transition dipole together with nearly perfect harmonicity, have an important consequence for THz-pumping an optical phonon in a solid. That is, even with modest field strengths, one excites a coherent state with a very large average phonon number (on the order of 10^6^) or, in other words, one climbs the vibrational ladder of the phonon by very many steps in a multi-photon process. Coherent optical phonons behave classically to a very good approximation, and thus the concept of a transition dipole moment $\left\langle i\left|\hat{\mu }\right|j\right\rangle$ between two quantum states *i* and *j* of a phonon, *per se*, is no longer a relevant parameter. Instead, the dynamics are often described in terms of the continuous development of a vibrational coordinate *Q* that follows a Newtonian equation of motion in response to the driving force of the THz field.

From the preceding arguments, it is clear that in some respects optical phonons behave differently from molecular vibrations, the latter of which are largely localized and often very anharmonic. That is in particular true for the intermolecular modes in liquids. But the scaling laws just introduced are such that for a given field strength *E* and a given local mode transition dipole *μ*_0_, the oscillation amplitude of individual atoms is in fact the same in both cases. In simple words, it makes no difference whether one is pumping many quanta into one particular optical phonon mode, or whether one is pumping one quantum into a large number of localized modes; in either case, the oscillation amplitude of individual atoms scales as *Eμ*_0_. For delocalized phonons, that can be seen when multiplying the phonon transition dipole ($\sqrt{n}\mu_{0}$) with the contribution of the individual unit cells ($1/\sqrt{n})$ to a phonon state. As we will see, the THz fields needed to enter a nonlinear (anharmonic) regime are very similar in both cases.

**FIG. 2.**
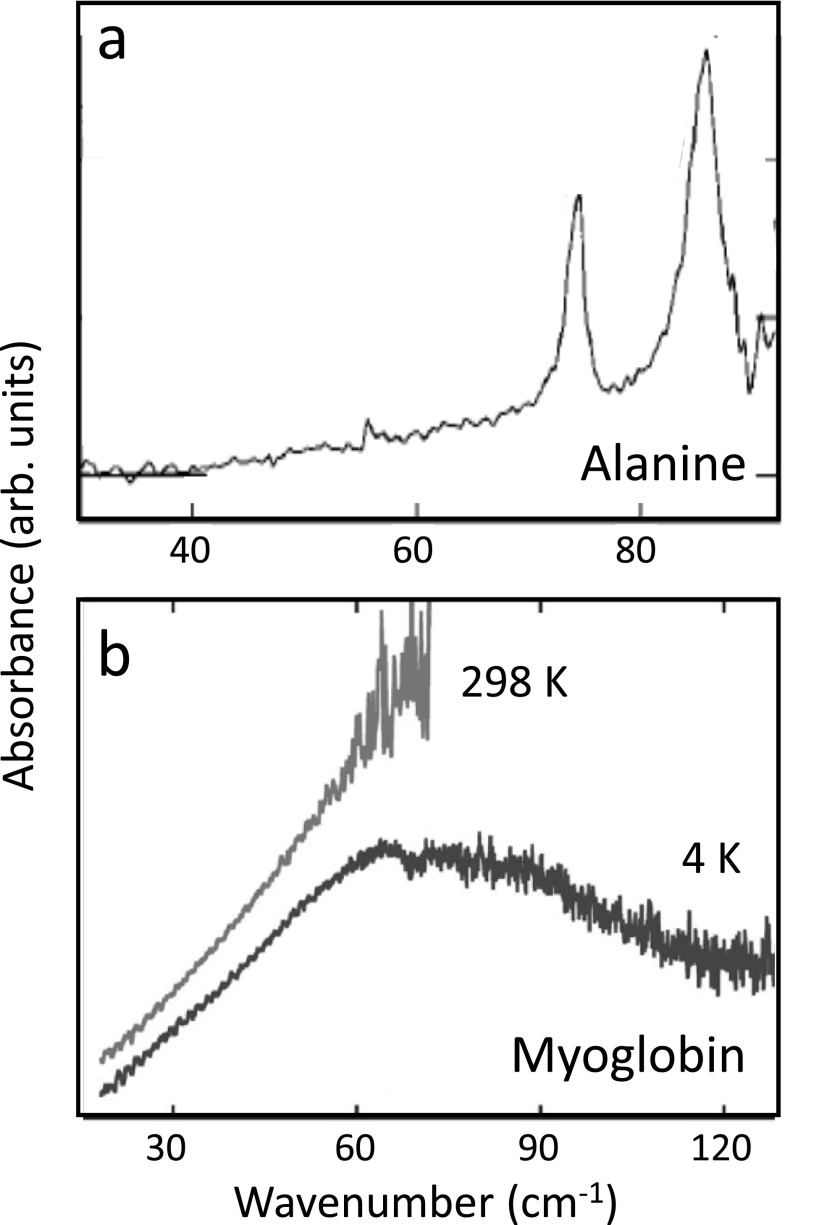
THz absorption spectra of (a) polycrystalline alanine at room-temperature and (b) myoglobin at 4 K as well as room-temperature. Panel (a) is reproduced with permission from Yamaguchi *et al.*, Appl. Phys. Lett. **86**, 53903 (2005). Copyright 2005 AIP Publishing, and panel (b) is reproduced with permission from Plusquellic *et al.*, ChemPhysChem **8**, 2412 (2007). Copyright 2007 Wiley VCH.

Despite these similarities, there are some very important differences between crystalline and more disordered molecular systems. On the one hand, in particular, soft mode excitations in solids are often connected with pronounced charge relocations on length scales that are much longer than the vibrational elongations.^74^ On the other hand, the large amount of anharmonicity and disorder in non-crystalline molecular systems leads to a dramatically higher level of damping. That is highlighted in Fig. 2, which compares THz absorption spectra of (poly)crystalline alanine,^75^ one of the simplest amino acids, with that of myoglobin,^76^ a small α-helical protein. Molecular crystals of amino acids are sometimes considered models for proteins, but Fig. 2 clearly emphasizes that this is not correct in the context of THz spectroscopy. That is, a molecular crystal of amino-acids still exhibits reasonably sharp absorption lines, which (at least for small amino acids such as alanine) are believed to be mostly of intermolecular character: phonons in the language of solid state physics. Any spectroscopic feature of that sort disappears in the protein, despite the fact that α-helices are repeat units with some degree of regularity and translational symmetry. Both disorder due to the structural complexity and very fast dephasing smear out the spectroscopy, but 2D spectroscopy has the potential to disentangle these effects to a certain extent (see below).

### B. Electrical field strength of typical THz pulses

The other factor that determines the size of the perturbation that one induces with a THz pulse is the electrical field strength *E* in its focus, which we can try to maximize as an experimentalist in order to make a nonlinear THz or a THz-pump experiment feasible. The electrical field strength of a laser pulse scales as $E\propto \sqrt{I}=\sqrt{W/\Delta td^{2}}$, where *I* is the intensity of the pulse, *W* is its energy, Δ*t* is the pulse duration, and *d* is the diameter of the laser focus. None of these factors are favorable in the THz regime,^77^ i.e., the pulse length is limited by the length of one optical light cycle (1 ps at 1 THz), the focus diameter by the wavelength of the pulse (300 *μ*m at 1 THz), and the conversion efficiency from the near IR (NIR) into the THz regime is low.

One of the most common sources of THz pulses is the optical rectification of a short NIR-pulse in a nonlinear crystal.^78^ Still by far the most widely used crystal for that purpose is ZnTe, whose THz conversion efficiency, however, is only 10^−6^–10^−5^, and hence, the maximal field strengths that can be achieved with that crystal are modest (a few 10's of kV/cm).^79,80^ With recent developments using other nonlinear crystals, in particular LiNbO_3_ ^81–85^ and organic nonlinear crystals such as DAST, OH1, or DSTMS,^86–88^ the situation improved significantly, reaching field strengths as high as 1 MV/cm in LiNbO_3_^82,85^ or even 80 MV/cm in DSTMS.^87^ Despite the larger conversion efficiency of these crystals, such high field strengths still require very large pulse energies for the NIR pump pulses, and hence are available only at relatively low repetition rates (10–100 Hz).^81,87^ Furthermore, while a very simple collinear geometry can be used for ZnTe when pumping at the Ti:Sapphire wavelength of 800 nm, the nonlinear rectification process is not phase-matched in LiNbO_3_ or in any one of the organic crystals. Therefore, one pumps LiNbO_3_ with a tilted pulse front in order to match the group velocity of the optical pump with the phase velocity of the THz beam,^89^ or one needs an extra optical parametrical amplifier (OPA) to shift the pump wavelength farer into the NIR for DAST, OH1, or DSTMS. Other sources of intense THz pulses are plasma sources with reported field strengths ranging from 400 kV/cm to 1.4 MV/cm,^90–92^ or electron accelerator-based sources (up to 20 MV/cm).^93,94^

The light sources mentioned so far produce essentially single-cycle pulses that are spectrally very broad. The ideal bandwidth of a pulse for exciting a nonlinear process depends on the characteristics of the material and the frequency and bandwidth of its modes. Soft modes in solids are in general fairly broad; hence, for driving soft-mode-mediated transitions, these are appropriate THz sources. That statement holds even more so for molecular systems, where the THz spectra are often completely smeared out, see e.g., Fig. 2(b). Furthermore, in the emerging field of 2D THz spectroscopy, one desires an impulsive excitation with ideally a pulse, which is shorter than any timescale of the system under study and which spectrally covers all its THz transitions. Some experiments in solids, however, require a high level of spectral power in a narrow bandwidth to selectively excite “hard” vibrational modes. For this purpose, difference frequency mixing in GaSe can also be used to generate a narrower bandwidth THz pulse over a range of frequencies from 15 THz to beyond 30 THz. In this regard, field strengths up to 100 MV/cm have been reported at 30 THz.^95^ More recent work makes use of chirped pulse difference frequency generation,^96^ with reported field strengths as high as 3.5 MV/cm in narrow-band pulses with frequencies ranging between 4 and 18 THz.^97^ Review articles focusing on the generation and detection of THz pulses can be found in Refs. 36–38.

It is important to realize that all these methods, in particular, optical rectification and difference frequency mixing in a nonlinear crystal, produce intrinsically carrier-envelope-phase stable THz pulses. Repeated excitation with these pulses in a pump-probe experiment maintains not only the temporal intensity profile but also the phase of the electric field, thereby enabling coherent excitations of the charges, to which the THz fields couple.

**FIG. 3.**
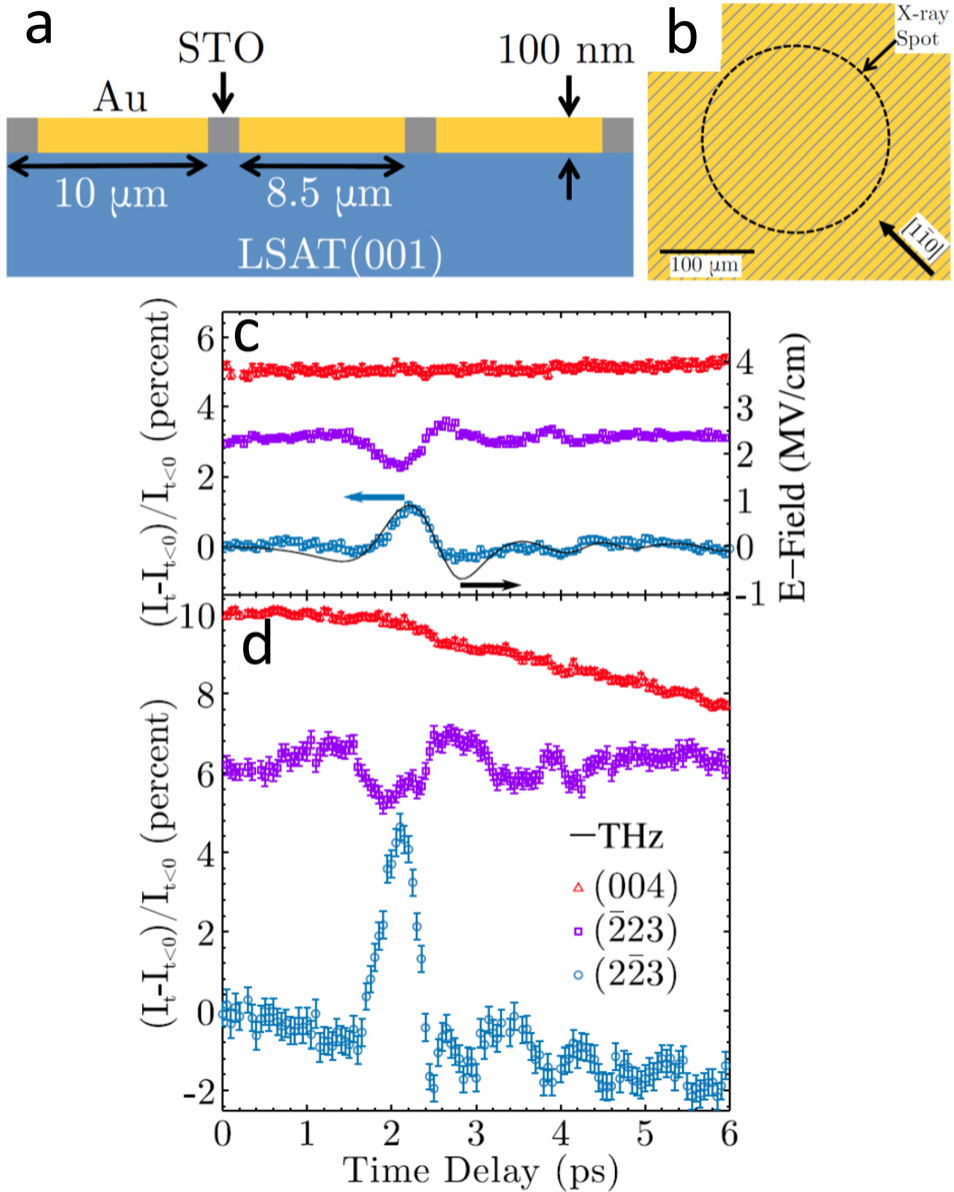
THz-pump-X-ray-probe experiment of SrTiO_3_ (STO). (a) Schematic cross-section of the sample and (b) the surface of the STO sample between Au stripes used for the field enhancement on a [001] (La_0.3_Sr_0.7_)(Al_0.65_Ta_0.35_)O_3_ (LSAT) substrate. The gold stripes are oriented parallel to [110]. The dashed circle represents the X-ray spot on the sample at normal incidence. Panels (c) and (d) show time-resolved Bragg peak intensity changes for the (004), (223), and (223) diffraction peaks (red triangles, purple squares, blue dots, respectively) at 100 K and 900 kV/cm, corresponding to ∼200 kV/cm inside the film, for the bare sample (c) and the sample with metamaterial (d). In the second case, the THz field and correspondingly also the amplitude of the phonons are enhanced by roughly a factor 5. Reproduced with permission from Kozina *et al.*, Appl. Phys. Lett. **110**, 081106 (2017). Copyright 2017 AIP Publishing LLC.

A complementary route to generate large THz field strengths uses near field enhancement effects in resonant metallic structures or in metamaterials acting as antennas,^12,98–100^ with demonstrated enhancement factors up to 30.^100^ In order to make use of enhanced fields in a THz-pump-X-ray probe experiment, it is essential that the THz temporal structure remain unchanged and that the metamaterial design does not interfere with the X-ray measurement geometry. Recent studies therefore used metamaterial designs that incorporated patterns of a large area of thin film samples with metal stripes several micrometers in width and several hundred nanometers thick.^12,101^ This allows probing with an X-ray spot size that is much larger than the stripe period. Furthermore, it is advantageous to remove part of the sample below the metal stripes to ensure that scattering from the sample comes only from regions excited by the THz pulse. This overall approach has recently been demonstrated for the measurement of coherently excited phonons in a thin film of SrTiO_3_ (STO),^12^ in which the X-ray penetration depth was closely matched to the depth of the THz field enhancement provided by the metal stripes [see Figs. 3(a) and 3(b)]. In this experiment, the THz field has been enhanced by roughly a factor 5 reaching 1 MV/cm, and correspondingly, the amplitude of the impulsively excited phonons has been enhanced by the same factor [Figs. 3(c) and 3(d)].

To put the THz field strengths reported above into relation, one may compare them with the intrinsic fields inside solids or molecules. For examples, the intra-atomic electrostatic field for the electron in an hydrogen atom is 5 GV/cm at the Bohr radius, and local fields strengths in solution or in complex molecular environments such as proteins or inside a biological membrane can be as high as a few 10's to 100 MV/cm.^102–104^ For solid-state ferroelectic materials, the local atomic fields can be estimated using the Mosotti relation as *P*/3ε_0_, where *P* is the spontaneous polarization. In BaTiO_3_, this comes to approximately 60 MV/cm.^105^

### C. Interaction energy

It is illustrative to put the interaction energy *μΕ* in relation to other quantities of the system. For example, as a measure of the amount of excitation that can be achieved, it is common to relate the resulting Rabi frequency *μΕ*/*h* to the length of the THz pulse.^54–57,68,77^ Taking the interband transition dipole of InSb as an example (190 D)^55^ and assuming an electrical field strength of 10 kV/cm from a ZnTe crystal,^79,80^ one obtains a Rabi frequency of 1 THz, i.e., one can saturate the transition with a typical single-cycle THz pulse. For a two level system, which electronic transitions often are to a reasonably good approximation, saturation also implies a nonlinear THz response: in the extreme case of π/2 or π-pulses beyond the perturbative limit.^55^ To achieve the same for a vibrational transition (0.5 D), a THz pulse with 4 MV/cm would be required, which is not out of reach, as discussed above,^87^ but technologically significantly more demanding. It is important to remember that one cannot saturate a vibrational mode when the harmonic approximation applies and that there is no nonlinear response, but the estimate nevertheless tells that the mode is excited to its first excited state or beyond. On the other hand, if a vibrational mode is strongly anharmonic, that estimate also gives the THz field strength at which one enters a non-linear regime.

**FIG. 4.**
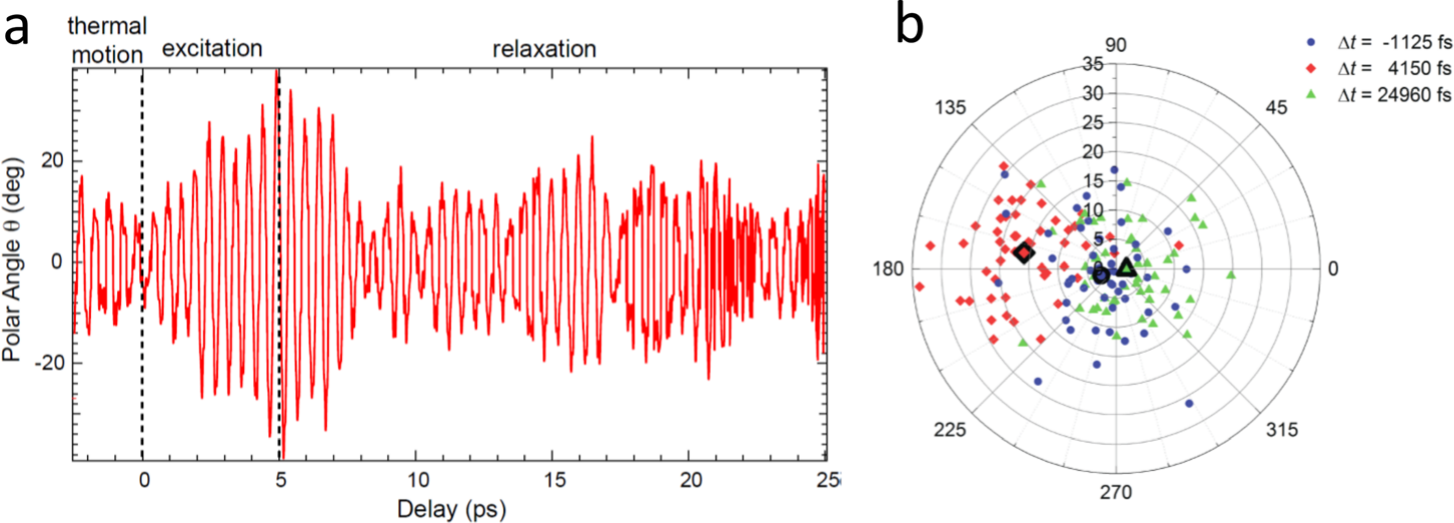
MD simulation of the wagging mode of a CO molecule bound to a Pt-surface before, during, and after resonant THz excitation. Panel (a) shows the amplitude from a single MD simulation trajectory and panel (b) the oscillation amplitude and phase of a distribution of trajectories at the indicated times. Data points highlighted with bold lines represent the centers of gravity of the respective distribution. The amplitude before time 0 represents the thermal noise, it rises during the THz pulse between 0 and 5 ps by a factor of ∼2, and then decays back to thermal noise on a ∼5 ps timescale. Reproduced with permission from Greif *et al.*, Struct. Dyn. **2**, 035102 (2015). Copyright 2015 AIP Publishing LLC.

The other parameter with which the interaction energy *μΕ* can be compared is thermal energy *k*_B_*T*. At room temperature, *k*_B_*T*/*h* is ∼6 THz. In order to perturb a system beyond thermal noise, and as such to enter a nonlinear regime, the interaction energy should be in that range. Thermal energy is of about the same order of magnitude as the typical THz frequency itself, which is a major motivation for nonlinear THz spectroscopy in the first place. Hence, both the Rabi frequency and thermal energy reveal about the same estimate for the anticipated interaction energy *μΕ*. A molecular dynamics (MD) simulation study illustrating this point has been published recently.^39^ In this work, a THz pulse with an assumed field strength of 12 MV/cm was used to excite the wagging mode of a CO molecule bound to a Pt surface. With the parameters of the simulation set-up, that mode has a transition dipole of 0.6 D, revealing *μΕ* = 0.6 *k*_B_*T*. Since the THz pulse is resonant and relatively long in this case (5 ps), the oscillation amplitude of the CO wagging mode reaches a level after the pump pulse that exceeds thermal noise at 300 K by about a factor 2 (see Fig. 4). After the pump-pulse is over, the oscillation amplitude decays back to thermal noise. The primary goal of Ref. 39 has been to explore the feasibility of THz-pump-photoelectron-diffraction-probe experiments of surface-bound molecules, which are becoming possible with the availability of ultrashort X-ray pulses from free-electron lasers.^34^

## III. FROM ELECTRONIC TRANSITIONS IN SOLIDS TO VIBRATIONS IN MOLECULES

### A. Linear and nonlinear phononics

Given the huge transitions dipoles to low-lying electronic states in solids, which make it relatively easy to reach the interaction energies just discussed with readily available THz pulses with field strengths in the order of 10–100 kV/cm, it can be understood why by far the most nonlinear THz experiments have been performed on such transitions, e.g., in semiconductors,^55–58,60,68,106–108^ superconductors,^109–112^ or graphene.^54,113–118^ For example, a Special Issue on nonlinear THz studies has been published recently (Ref. 77 and references therein), which presented exclusively experiments exciting low-lying electronic transitions in solids with a THz pulse.^60,68,106–108,110–112,116,117^

**FIG. 5.**
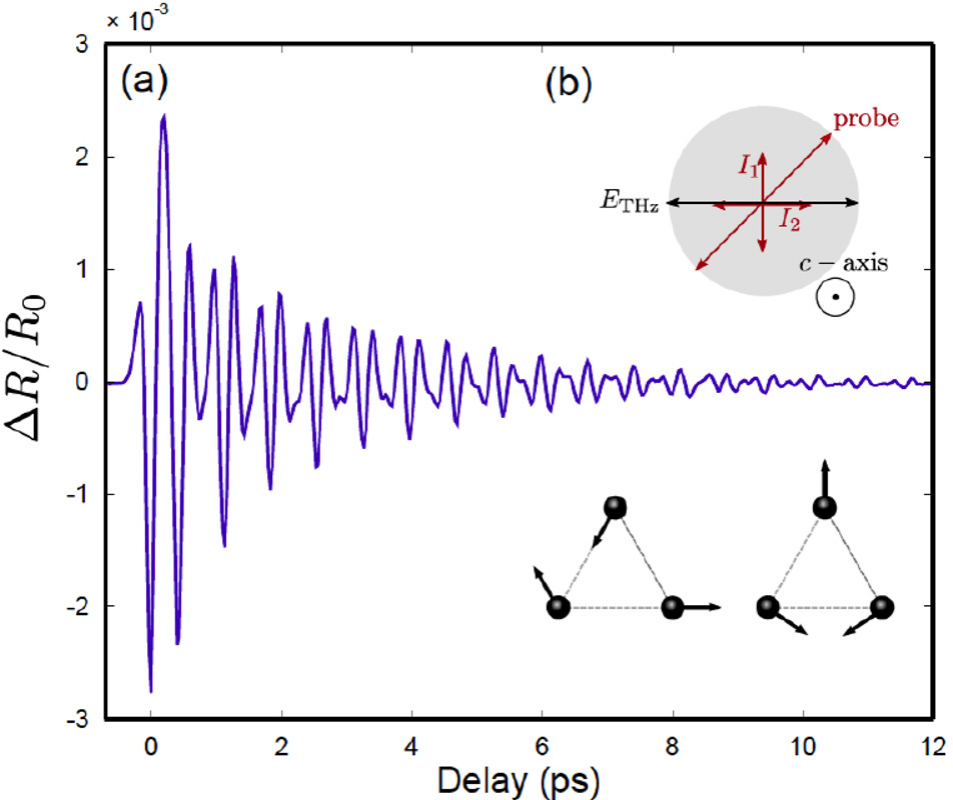
Changes in the anisotropic reflectivity caused by the *E*-symmetry lattice modes in a Te-crystal impulsively driven by a single-cycle THz pump pulse. A Fourier transformation of the signal reveals two IR active phonons of *E*-symmetry, which are indicated in the inset. Reproduced with permission from Huber *et al.*, Appl. Phys. Lett. **107**, 091107 (2015). Copyright 2015 AIP Publishing LLC.

Quite recently, THz experiments exciting phonons have appeared in literature,^10–13,41–43,46^ which closely resemble the character of molecular vibrations, i.e., where the transition dipole is determined by the fractional (partial) charges of the nuclei that are considered approximately constant as a function of the atomic displacements, and are not directly coupled to electronic excitation of the system. Figure 5 shows a proof-of-principle example in this regard. A broadband THz pulse impulsively drives a number of different vibrational modes in a single crystal of tellurium with frequencies within the bandwidth of the driving field.^41^ The nonlinear response of the lattice is small in this case. The excited coherent modes are detected via time-dependent changes to the reflectivity of the crystal surface in the near-infrared part of the electromagnetic spectrum, probed by short femtosecond duration pulses centered at 800 nm wavelength. The results show clear responses from two pairs of *E*-symmetry vibrational modes that correlate well with expectations from a simple Lorentz oscillator model of the lattice dynamics. Along the same lines, phonons have also been excited in SrTiO_3_^12,13^ by THz fields when driving the polar soft modes of the crystal, in this case, measuring the response by time-resolved X-ray diffraction that directly reports on the displacement of the lattice atoms as a function of time.

**FIG. 6.**
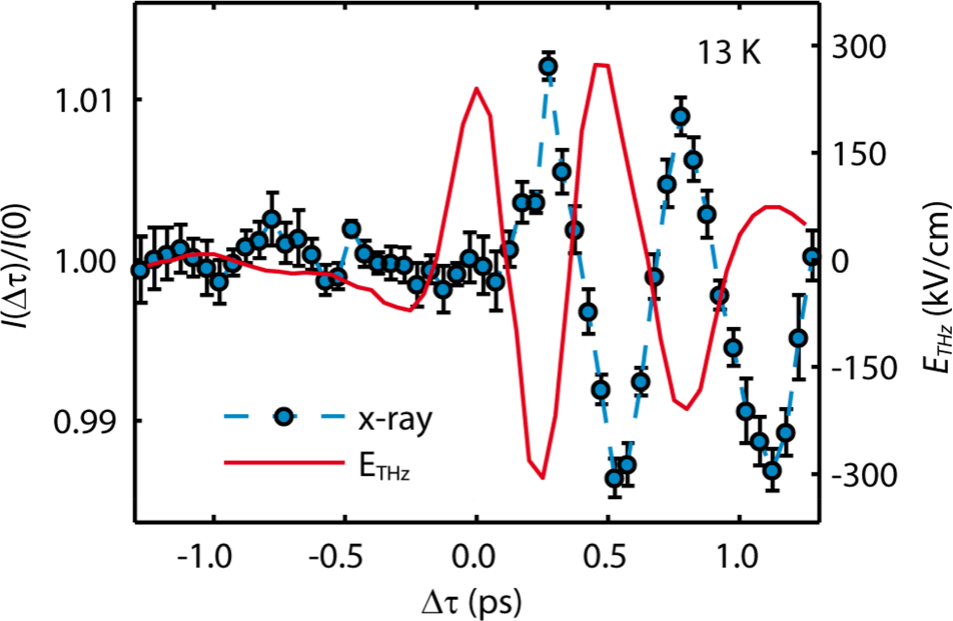
Magnetic diffraction intensity *I* of the (0q0) peak of TbMnO_3_ (blue symbols, left axis) compared with the electric field *E*_THZ_ of the pump trace (red solid line, right axis) as a function of the time delay. Reproduced with permission from Kubacka *et al.*, Science **343**, 1333 (2014). Copyright 2014 The American Association for the Advancement of Science.

A more involved example in this regard are electromagnons, which have first been observed in TbMnO_3_,^119^ a material where the electric and magnetic degrees of freedom are intrinsically coupled in its multiferroic phase. These magnetic modes are a mixture of phonons and magnons, which allows them to be excited with the *E*-field of a THz pulse. In this phase, the material obtains a polarization due to the cycloidal Mn spin order that breaks the inversion symmetry of the system.^120^ It has been theoretically predicted that excitation of the electromagnon with a single cycle pulse of several MV/cm field strength will lead to a spin motion that reverses the spin rotation of the cycloid structure.^50^ Such a reversal would not only switch the antiferromagnetic domain, but would also result in a reversal of the polarization of the material. A recent experimental study has been able to show that the concept behind the theoretical work is indeed valid.^10^ Using a low cycle THz pulse, the electromagnon was excited and the time dependence of the magnetic structure could be followed using ultrashort X-ray pulses (Fig. 6). Even though the directly driven magnetic mode could not be observed in this set-up, the observed spin motion is consistent with the secondary motion of rotating the cycloid, as predicted by theory. The experiment used THz excitation pulses with a field strength of 65 kV/cm inside the sample and resulted in a spin-cycloid plane rotation of 4.2°, strengthening the idea that a reversal of the magnetic cycloid should indeed be possible for sufficiently large THz fields.

In all these examples,^10,12,13,41,121^ the system response remained in a linear regime as a function of applied THz field strengths. The ultimate motivation of many of these works is to enter a nonlinear (anharmonic) regime,^46^ which in the extreme case will result in a phase transition. For example, it has been estimated in Ref. 10 that field strengths higher than 1–2 MV/cm would be needed for a complete spin reversal of the electromagnon in TbMnO_3_. Field strengths in that order of magnitude are within reach for today's THz sources,^87^ or when enhancing the THz field with the help of metamaterials.^12^ Similarly, THz fields of ∼1–5 MV/cm have been estimated as needed to drive a polarization reversal in ferroelectric materials such as SrTiO_3_^46^ or PbTiO_3_.^121^

**FIG. 7.**
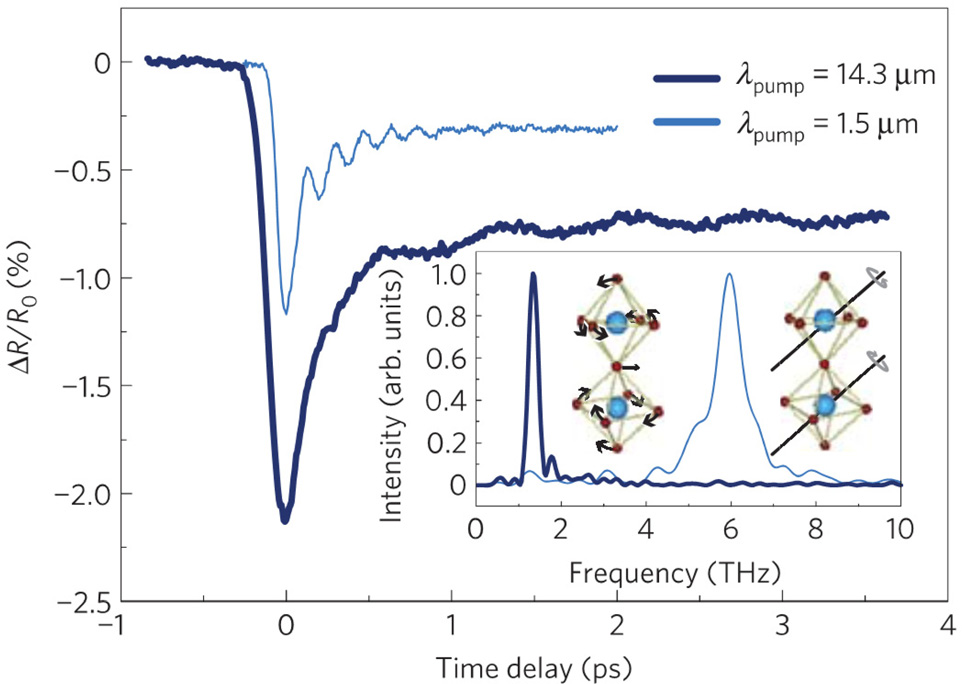
Time-dependent reflectivity from single crystal La_0.7_Sr_0.3_MnO_3_ as a function of time with respect to femtosecond duration pump pulses at 1.5 *μ*m (dark blue) and 14.3 *μ*m (light blue). The inset shows Fourier Transforms of the oscillatory part of the traces, as well as sketches of the eigenvectors corresponding to the phonon modes close to where the experiment observes frequency maxima. Reproduced with permission from Först *et al.*, Nat. Phys. **7**, 854 (2011). Copyright 2011 Nature Publishing Group.

One way to access a more strongly nonlinear regime in solid state materials is to focus more on driving large-scale dynamics in higher frequency vibrational modes where damping is less strong. This is a strategy that has led to the discovery of several time-domain manifestations of nonlinear phenomena, now broadly called nonlinear phononics.^51,122,123^ One of the more basic ideas of such experiments is exemplified by the work of Först *et al.* shown in Fig. 7.^44^ In this work, a conventional near-infrared pump (1.5 *μ*m) first electronically excites dynamics in La_0.7_Sr_0.3_MnO_3_, which are then probed by measuring the time-dependent reflectivity. The light blue curve in Fig. 7 shows the response, which is characterized by a drop in the reflectivity followed by a partial recovery with oscillations at a frequency of about 6 THz superimposed on the recovery. These oscillations correspond to an optical phonon mode in the crystal that is depicted in the inset of Fig. 7 on the right: essentially, a coordinated rotation of the oxygen octahedral that surrounds the Mn sites in the crystal lattice. This is an example of a fairly widespread phenomenon in pump-probe spectroscopy known as displacive excitation of coherent phonons (DECP).^124^ Essentially, the near-infrared pulse drives a change in the occupation of electronic states, which then couples to the vibrational mode as a displacement of its quasi-equilibrium displacement. The result is a coherent oscillation of the vibrational mode around its new quasi-equilibrium value.

The situation changes, however, when the pump pulse wavelength is tuned to a wavelength of 14.3 *μ*m, a wavelength close to resonance with a vibrational mode at 20 THz characterized by a motion of the oxygen ions against the Mn ion. The result is also shown in Fig. 7 by the dark blue line. The drop and partial recovery are similar, but the frequency of the oscillation is now at about 1.5 THz, indicating that a different vibrational mode is excited. This mode corresponds to a twisting of the Mn-O octahedral as shown on the left side of the inset. This response is seen only when the pump is tuned very close to the frequency of the 20 THz vibrational mode, which is strong evidence that the observed coherent phonon mode at 1.5 THz is driven via anharmonic coupling terms by the excitation of the 20 THz phonon mode, in analogy to the nonlinear coupling of light in nonlinear optical materials. More specifically, the 1.5 THz mode is driven to leading order by an anharmonic term in the vibrational part of the system Hamiltonian with the form Q_1_Q_2_^2^, where Q_1_ is the coordinate of the 1.5 THz (Raman-active) mode and Q_2_ is the coordinate of the 20 THz (IR-active) mode.

Although the values of these indirectly excited anharmonically coupled modes are usually fairly small, they can sometimes lead to large changes in the electronic properties of materials, particularly in the case of strong correlated systems where there are many competing interactions at play. For example, the vibrational mode excitation of mixed-valence manganites and nickelates has driven large changes in electrical conductivity and magnetic order.^119,125–128^ There have also been several recent experiments on superconducting materials that show the evidence of superconducting-like behavior that can be induced by excitation of vibrational modes.^18,51,129,130^ Nonlinear phononic effects can also create transient magnetic fields, thereby exciting magnetic excitations.^131^ In the context of control over ferroelectrics, there is even a recent experiment that sees some indications of possible nonlinear-phononics-induced transient reversal.^132^ For interested readers, who would like to know more on this subject, we refer to some recently published review articles in Refs. 51, 122, and 123.

Nonlinear phononics is, however, not the only way in which vibrational pumping has produced nonlinear responses in crystalline materials. Large-amplitude vibrational modes can also couple directly to the electronic degrees of freedom of the material, without the need to act through an auxiliary phonon mode.^133^ In a recent study of the vibrationally excited manganite Pr_0.5_Ca_0.5_MnO_3_, a quartic dependence of the gap closure on the amplitude of the excited phonon was found to be responsible for the induced insulator-metal transition.^20^

### B. Molecular systems

For molecular systems without long-range translational order, the number of nonlinear THz experiments pursued as of today is rather limited. Nelson and co-workers have pioneered nonlinear THz experiments on molecular systems with what became known as “THz Kerr effect.”^47^ In these experiments, which have been demonstrated both in the gas^134,135^ and in the solution phase,^47–49^ an intense THz pulse aligns molecules by acting on their orientational polarizability, and a NIR-probe pulse measures the induced birefringence via a Raman interaction. These experiments are intrinsically nonlinear in the THz field, scaling as $E_{THZ}^{2}E_{NIR}$, since otherwise they would rest on a χ^(2)^-effect (scaling as $E_{THZ}E_{NIR}$) that is forbidden in an isotropic sample. The quadratic dependence on the THz field, in turn, is the basis for an extension of this experiment toward a hybrid 2D-THz-Raman-spectroscopy. That is, rather than taking the two THz field interactions from one and the same THz pulse, one applies two THz pulses separated by a second controllable time delay in addition to the time delay of the Raman-probe pulse. Both delay times are coherence times and thus may be 2D Fourier-transformed to reveal a 2D spectrum.^136,137^ Furthermore, since this method involves both THz and Raman interactions, interchanging the time-ordering of these processes reveals variants of the method, and both the Raman-THz-THz and THz-Raman-THz pulse sequences have been explored as well.^138,140^ These experiments involve a Raman and a THz pump pulse, and the response of the sample is read out via the emission of a THz field. A recent review on hybrid 2D-Raman-THz spectroscopy can be found in Ref. 141.

**FIG. 8.**
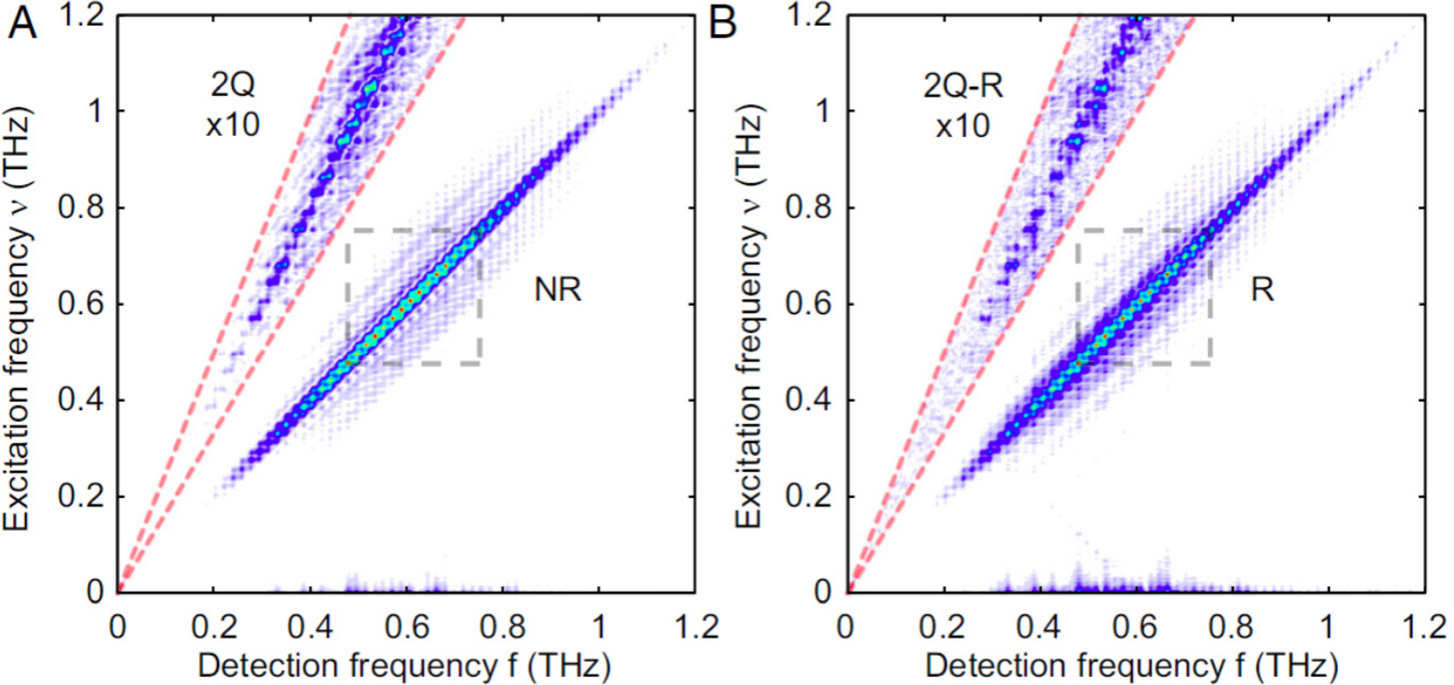
2D-THz spectrum of the rotational spectrum of CH_3_CN at room temperature. Panel (a) shows the non-rephasing (NR) quadrant and panel (b) the rephrasing (R) quadrant. In each case, two-quantum (2Q) transitions can be identified as well at lower intensity. Reproduced with permission from Lu *et al.*, Proc. Natl Acad. Sci. U. S. A. **113**, 11800 (2016). Copyright 2016 National Academy of Sciences.

The first, and as of today only, true 2D-THz experiment on a molecular system, i.e., with all pulse interactions in the THz regime, has been published recently by Nelson and co-workers, investigating the thermally excited rotational states of a polar molecule (CH_3_CN) in the gas phase, see Fig. 8.^59^ The 2D-THz spectrum is strongly elongated along the diagonal, which is a hallmark of inhomogenous broadening.^62^ In case of Fig. 8, the origin of the inhomogeneous broadening is the many rotational states that are thermally excited at room temperature. Similar experiments have been performed at even lower frequency for larger molecules in the microwave regime (not with a laser-based sources of the radiation), albeit at very low rotational temperatures.^142,143^ In this regard, it is worthwhile noting that it has been the by a factor of ∼10 larger transition dipole moment of rotational transitions, as compared to vibrational modes [Figs. 1(b) and 1(c)], together with the intrinsic anharmonicity of rotational states, which facilitated this experiment.

**FIG. 9.**
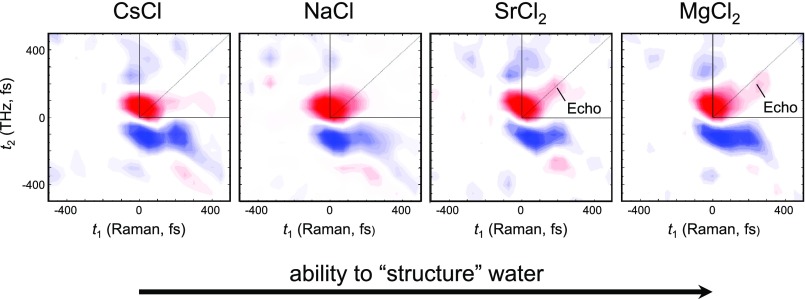
2D-Raman-THz time-domain responses of aqueous solutions of CsCl, NaCl, SrCl_2_, and MgCl_2_. The measurements are ordered along the ability of the cation to “structure” water. Cs^+^ is considered to be a “structure breaker,” while Sr^2+^ and Mg^2+^ are medium-strong “structure maker.”^145^ In the latter two cases, echoes appear along the diagonal with *t*_1_ = *t*_2_, indicating the spectral inhomogeneity in the low-frequency THz spectrum, which originates from the structural heterogeneity induced by the “structure-making” salt. Reproduced with permission from Shalit *et al.*, Nat. Chem. **9**, 273 (2017). Copyright 2017 Nature Publishing Group.

The questions asked by 2D-THz and 2D-Raman-THz spectroscopy address the electrical and mechanical anharmonicity of the investigated vibrational modes,^136^ the coherent control of molecular orientation,^59^ and the amount of inhomogenous broadening in liquids.^139,140^ For the latter, it is important to realize that typical THz absorption spectra of solution phase systems, and in particular, those of the liquids themselves, are extremely blurred, owing to the large anharmonicity and ultrafast dynamics of the intermolecular modes and the structural complexity of the liquid, which in fact severely limits the information content that may be retrieved from conventional THz spectroscopy. Sometimes, THz spectroscopy is therefore referred to as “blob spectroscopy,”^144^ see, e.g., Fig. 2(b) for a protein. Yet, the THz spectrum contains, in principle, all information on the intermolecular forces that are so responsible for the thermodynamic and dynamic properties of these systems. Trying to extract as much as possible information from such spectra calls for a multi-dimensional technique.^62^ 2D-Raman-THz spectra of liquid bromoform,^136,137^ liquid water,^139^ and of aqueous salt solutions^140^ have been measured with different time-orderings of Raman and THz interactions. The latter experiment^140^ reveals an echo for salts that are characterized as so-called “structure-makers” (see Fig. 9).^145^ The very concept of echoes in spectroscopy has been introduced a long time ago first for NMR,^146^ and later for both electronic^147^ and vibrational transitions.^148,149^ Echoes appear when the transition under study is inhomogeneously broadened, in this particular case, due to water hydrogen-networks that are stabilized on a certain timescale by the presence of structure-making salts.^141^

## IV. CONCLUSION

While experiments using intense THz pulses to excite low-lying electronic states in strongly correlated materials are quite standard nowadays, easily reaching a non-linear and even a non-perturbative regime with rather modest THz field-strengths, the same is not yet true when triggering nuclear motion directly with intense THz pulses. The electric field of THz pulses couple most directly to the charges of the nuclei, thereby opening a window to a fundamentally new approach to control the structure of solid state materials and molecular systems. However, these experiments are experimentally significantly more demanding, since the transitions dipoles of the involved states are smaller by typically two orders of magnitudes (Fig. 1). Recent technological developments in THz pulse generation now are reaching field strengths that are capable of compensating for these smaller transition dipoles. Effective enhancement of the THz field by metamaterials is an alternative route with a great potential for nonlinear THz experiments.

It is interesting to note that regarding the transition dipole, there is no fundamental difference between phonons in a solid and vibrations in an isolated molecule, despite the fact that at a first glance both behave very differently regarding their delocalization volume and anharmonicity. This similarity is evidenced by the estimates that have been given for the field strengths (1–10 MV/cm) needed to drive phonons into a strongly nonlinear regime, potentially inducing a phase transition,^10,20,46^ are of the same order of magnitude as those (4 MV/cm) needed to “saturate” the 0–1 transition of a vibration and to enter an anharmonic regime for an isolated molecule. The experimental challenges regarding the THz pulse generation are very similar in both cases.

The solid-state and molecular physics communities can spur each other, and also learn from one another. For example, while the very first 2D-THz experiment has been performed on intersubband transitions in an GaAs/AlGaAs multiple quantum well,^56^ facilitated by the huge dipole moment of these electronic states [see Fig. 1(a)], the extension of that technique to include vibrational excitations has remained relatively rare. The only example we are aware of is the work of Ref. 58, measuring the Coulomb-mediated interactions between intersubband excitations of GaAs/AlGaAs quantum wells and longitudinal optical phonons, where the phonon intensity was enhanced by strong polaronic effects. 2D-THz experiments on nuclear degrees of freedom are more developed in the molecular world, where numerous techniques have been applied to study a large variety of molecular systems and addressing different questions.^59,136–143^ In contrast, the solid state physics community is much more advanced in inducing phase transitions with THz pulses,^10,18,20,46,50,51,129^ while THz-induced phase transitions in liquids and soft matter materials will probably work via a thermal mechanism only due to the very rapid dissipation of energy in these materials. Experiments of the kind shown in Fig. 4, inducing structural changes in molecular systems, should become feasible now.^39^ The direct control of the orientation of molecules adsorbed to a catalytic surface, thereby inducing the catalytic reaction, would be a beautiful example of an especially direct realization of the very idea of femtochemistry.^1^
